# Expect delays: poor connections between rural and urban health systems challenge multidisciplinary care for rural Americans with diabetic foot ulcers

**DOI:** 10.1186/s13047-020-00395-y

**Published:** 2020-06-16

**Authors:** Bryn L. Sutherland, Kristen Pecanac, Christie M. Bartels, Meghan B. Brennan

**Affiliations:** 1 Department of Medicine, University of Wisconsin School of Medicine and Public Health (UWSMPH), 800 University Bay Dr. Suite 210, Madison, WI 53705-2299 USA; 2 School of Nursing, University of Wisconsin-Madison, 4167 Signe Skott Cooper Hall, 701 Highland Ave, Madison, WI 53705-2299 USA

**Keywords:** Diabetic foot, Health care delivery, Interdisciplinary studies, Patient care team, Rural health, Referral and consultation

## Abstract

**Background:**

Rural Americans with diabetic foot ulcers (DFUs) face a 50% increased risk of major amputation compared to their urban counterparts. We sought to identify health system barriers contributing to this disparity.

**Methods:**

We interviewed 44 participants involved in the care of rural patients with DFUs: 6 rural primary care providers (PCPs), 12 rural specialists, 12 urban specialists, 9 support staff, and 5 patients/caregivers. Directed content analysis was performed guided by a conceptual model describing how PCPs and specialists collaborate to care for shared patients.

**Results:**

Rural PCPs reported lack of training in wound care and quickly referred patients with DFUs to local podiatrists or wound care providers. Timely referrals to, and subsequent collaborations with, rural specialists were facilitated by professional connections. However, these connections often were lacking between rural providers and urban specialists, whose skills were needed to optimally treat patients with high acuity ulcers. Urban referrals, particularly to vascular surgery or infectious disease, were stymied by 1) time-consuming processes, 2) negative provider interactions, and 3) multiple, disconnected electronic health record systems. Such barriers ultimately detracted from rural PCPs’ ability to focus on medical management, as well as urban specialists’ ability to appropriately triage referrals due to lacking information. Subsequent collaboration between providers also suffered as a result.

**Conclusions:**

Poor connections across rural and urban healthcare systems was described as the primary health system barrier driving the rural disparity in major amputations. Future interventions focusing on mitigating this barrier could reduce the rural disparity in major amputations.

## Background

Over 30 million people in the United States have diabetes, and up to 25% of them will develop a diabetic foot ulcer (DFU) [1–3]. Within five years of ulceration, 5% of patients lose a limb and over 50% die [4–8]. Rural Americans, defined as the one in five people living in areas with fewer than 50,000 people, are particularly vulnerable to poor health outcomes [9–13]. Specific to DFUs, rural patients face 50% higher odds of major (above-ankle) amputation and 40% higher odds of death compared to their urban counterparts [9, 10]. We do not know what drives this health disparity.

The aim of our study was to understand what health system factors contribute to the rural disparity in DFU outcomes. We chose to focus on health system factors because 1) they are modifiable, 2) they may be generalized to other rural disparities, and 3) recent advances in urban health systems have reduced the risk of major amputations by ~ 40% [14]. Specifically, many urban health systems have initiated teams to provide multidisciplinary care that addresses four physiologic factors in a timely and coordinated manner: glycemic control, local wound management, vascular disease, and infection. These teams are composed of specialists working side-by-side within a single health system. Rural providers are unlikely to benefit from this model of multidisciplinary care due to the rarity of specialists and need to collaborate across multiple health systems.

We chose qualitative methodology to understand health system factors contributing to the rural disparity in DFU outcomes because little is known about how rural patients receive care for their ulcers. Naturalistic inquiry guides our qualitative methodology, in which people construct their own meaning and interpretations to events and processes that shape their reality [15]. Here, we use provider, patient, and caregiver interviews to compile rich descriptions of the current process of care and how it may be contributing to, or mitigating, this disparity. Our intent is that this information will inform future, system-based interventions to address this rural disparity.

## Participants and methods

### Participants

Three groups of study participants were recruited: 1) rural primary care providers (PCPs), 2) other healthcare specialists (e.g. specialty providers, nurses, allied health professionals, and administrative support staff), and 3) patients with DFUs/their caregivers. A purposive sample of rural PCPs (Group 1) was recruited through email solicitation with assistance from both the Wisconsin Research and Education Network (WREN) and the Rural Wisconsin Health Cooperative (RWHC). WREN is a voluntary network of over 50 primary care providers who engage in practice-based research. RWHC is an organization of 44 rural health systems dedicated to optimizing rural care through research, quality improvement, and advocacy. Rural healthcare specialists were identified through snowball sampling (Group 2).

Rural participants identified urban specialists as important to patient care but were unable to name specific urban specialists to whom they had referred during snowball sampling. Therefore, we hung flyers in urban, professional spaces and used our professional connections to identify urban specialists and recruit them (Group 2). Patients with DFUs and their caregivers were recruited by hanging flyers in various rural clinic sites (Group 3). All participants who expressed interest in the study gave verbal informed consent, including consent to be audio recorded, and completed the interview.

### Data collection

Each participant engaged in a single, hour-long, one-on-one interview. The researcher who conducted all interviews (B.S.) was a premedical team member from a rural community who had prior patient care exposure, but no preceding contact with study participants. The interviewer’s background allowed her to establish rapport and credibility without prior interactions influencing participant responses. The 14-item semi-structured interview guide was designed utilizing a conceptual model focused on collaborations between PCPs and specialists, modified to reflect components of DFU care (see Supplementary Information) [16]. The interview guide was piloted prior to use with a rural specialist, an urban PCP, and two urban specialists. These volunteers were not included as study participants. Input from RWHC and a qualitative methods research group informed iterative edits to the interview guide, allowing our team to pursue emerging themes in detail. All interviews were conducted in-person, except one that was conducted by phone. Every interview was audio recorded and transcribed verbatim for analysis. Participants received a $100 honorarium.

### Analysis

Directed content analysis was performed using categories/codes from the conceptual model that informed the interview guide [16, 17]. We also performed open coding to capture emerging themes that were absent from the initial model [18]. Two reviewers (M.B. and B.S.) independently coded transcripts and subsequently met to discuss discrepancies and arrive at consensus [19]. We modified our original conceptual model to reflect the care process described by our participants, with particular focus on health system barriers. The emerging model was member-checked with participants to ensure it reflected their experiences. We shared our results with RWHC members who were not interviewed, including quality improvement directors and systems leaders, to determine resonance with a broader audience. Lastly, the analysis was presented to an external qualitative research group to ensure methodologic rigor. All coding was performed using NVivo 12 (QRS International Inc., Burlington, MA).

## Results

We interviewed 44 participants with varied roles (Table 1). All six PCPs practiced in a rural setting spanning five unique health systems (Group 1). Seventeen of 33 non-PCPs worked in a rural setting (52%, Group 2); all five patients and caregivers lived in a rural setting (Group 3). All patients and caregivers had undergone, or assisted someone who had undergone, minor amputations due to diabetic foot ulcers.

**Table 1:** Participant roles and locations (*n* = 44)\

Participant role	Rural (n)	Urban (n)	Total (n)
Primary care	6	0	6
Clinical support (e.g. nurses, medical assistants)	4	1	5
Podiatry	3	3	6
Diabetes education	3	0	3
Wound specialist	3	0	3
Home health	2	0	2
Infectious disease	1	2	3
Administrative support (e.g. schedulers, referral coordinators)	1	3	4
Vascular surgery	0	5	5
Social work/case management	0	2	2
Patient	2	0	2
Caregiver	3	0	3
**Total**	28	16	44

**Fig. 1:**
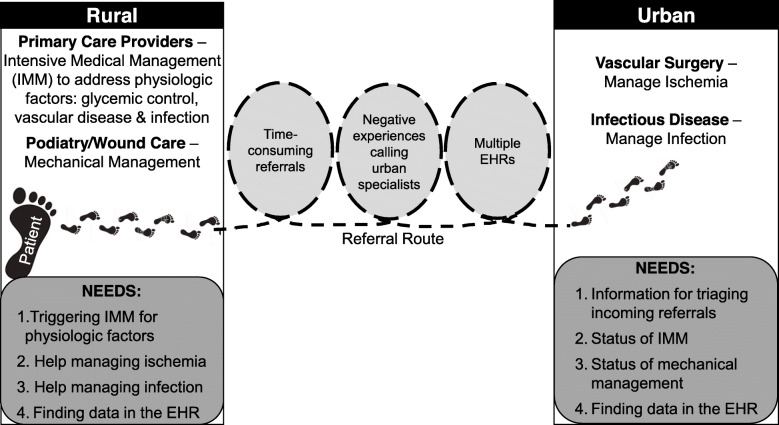
Conceptual model describing how rural patients receive care for diabetic foot ulcers

Our conceptual model of how rural patients receive care for DFUs identified referral to, and collaboration with, urban specialists as a prominent health system barrier (Fig. 1). Professional connections between providers within the same rural health system facilitated timely, multidisciplinary care. However, these connections were lacking between rural providers and urban specialists, who struggled to bridge information gaps between multiple health systems. These themes are explored in detail below, and Table 2 contains additional quotes supporting each.

**Table 2:** Key themes and supporting quotes

Theme	Supporting quotes
Rural PCP- rural specialist collaboration facilitated by professional connections	“In a small hospital, you know who the wound care nurse is. You can just call them up or email to touch base, or I’ll just stop by.” *Rural diabetes educator* “When I take off somebody’s shoe and see bone, I just walk over to the podiatrist and say, ‘Could you come take a look?’”*Rural PCP* “I’m fortunate to be good friends with one of the orthopedic surgeons here. If I feel like somebody needs to be seen quickly, I just call him up and he’s very good about that kind of stuff. The podiatrist we used to have in town was super about seeing people the same day.” *Rural PCP*
Time consuming referrals	“A few years ago, the patient would walk out the door with a [specialty] appointment in hand, which I think is better. Now they have to call up some referral coordinator and then it gets computerized somehow, and then the computer sends it, and somebody from the specialty clinic ends up calling the patient if their insurance is good enough and all that stuff is approved.” *Rural PCP* “It’s become a little bit more challenging over the last few years. Years ago. .. we used to make the appointments ourselves. Now we have another step where we have a referral department, which helps determine things like, ‘This person has this type of insurance. They need to go here.’ Sometimes the process takes longer. You can’t call [a tertiary care hospital] anymore and say, ‘Hey I have this patient.. .’ You’ve got to send information, and they triage it. The process takes longer than it used to.” *Rural PCP* “The case manager is responsible for making sure that the physician order got entered for the referral and then we route the task to our receptionist. She processes it. She faxes the referral order and the physician’s notes and the imaging. We put that all in the referral order.. . Then she sends a face sheet and the referral order to the specialty provider, and she just puts on the cover sheet, ‘Please contact patient to schedule.’”*Rural administrative support staff* “It is time consuming to get the records. Sometimes the medical records departments at the other healthcare facilities don’t know exactly what we want. We have to be very specific with what we want.” *Urban administrative support staff* “By the time they come to us, they’re already at quite an advanced stage. So if we wait for those referrals to happen from the primary care provider and go through insurance coverage and this and that. . . There’s just too many loopholes.” *Urban infectious disease physician*
Negative experiences calling urban specialists	“I typically like to have all my ducks in a row before I call a specialist because I feel like family doctors are not really all that highly respected amongst the medical field.” *Rural PCP* “Doctors sometimes eat their young. Sometimes they are awfully critical of what others are doing.” *Rural PCP*
Multiple EHRs as a barrier to data sharing	“Somebody may have had a lab drawn in a different system. .. but I might not take the time to look because it would be a lot of work. It would be nice if lab and imaging results showed up in our EHR from other systems. It’s one of my biggest gripes. You can access results, but it would be nice if they just showed up automatically.” *Rural PCP* “We have different electronic medical records. We get patients from all over. Trying to gather all that information- I’m surprised these nurses have any hair left on their heads after trying to organize that stuff.” *Rural wound care provider* “Sometimes there are things in [the system that links two EHRs] that we cannot see, even though it will be in the system.” *Urban administrative support staff* “With the different places I go, there are different electronic medical records. Typically, I will look through to find as much information as I can on the patient before going to see them.. . Sometimes it’s very difficult.” *Urban vascular surgeon who does rural outreach*
The value of multidisciplinary care and teamwork	“The team is what makes us work. It can work just as well in a rural community as it does in the city, and I think it is maybe even more important.” *Rural wound care provider* “It is absolutely essential to try and conform our management plan within the realm of local resources. That’s very important― to have the local communities be part of the team.. . All of us have a role to play and something to contribute.” *Urban infectious disease specialist*

### Rural PCP- rural specialist collaboration

All six rural PCPs referred patients with DFUs to specialists because they lacked training in wound care and debridement: only one felt confident managing “very superficial” ulcers. PCPs typically referred to podiatry or wound care clinics, depending on which was locally available. Rural PCPs identified care coordination as their predominant role for patients with DFUs: “We are on the frontline for the patient, as their advocate. Especially in a rural setting, patients are so dependent on us to coordinate their care.”

Most PCPs relied on their professional connections with, and close proximity to, rural specialists when expediting referrals. For instance, the majority had working relationships with local podiatrists or wound care teams. They telephoned colleagues, shared wound pictures over secure messaging applications, and spoke face-to-face with rural specialists to facilitate urgent, outpatient care. One PCP described working with a wound care specialist: “They’re in the other pod, but you can walk over and say, ‘Hey, would you come look at this?’ And they’re willing to come over.. .That’s the nice thing about being in a small system.” Another recalled “[T] he best thing was when I happen to catch the podiatrist on rounds and we looked at the wound together, and talked to the patient about our plans. That was the most helpful because then the patient actually understood both of our perspectives.. . Now that was a perfect world.” While uncommon, both PCPs and specialists agreed that simultaneous, multidisciplinary care stood out as instances of “the best” care. Even when PCP efforts did not result in simultaneous, multidisciplinary care they almost always yielded timely consultations from rural-based specialists.

Close coordination and collaboration relied on professional connections, rather than healthcare systems. When these connections were not present, rural PCPs struggled to get the help their patients needed. One PCP who recently joined a rural practice stated:

Where do I send someone with a diabetic foot ulcer that’s beyond my skill level? Maybe it’s not an emergency, but they need a higher level of care. For one patient, I tried to do a general surgery consult, but they came back the next day and said, ‘No, our wound care nurses don’t see patients with diabetic foot ulcers. Try podiatry.’ There was an insurance issue with the first podiatrist, so we went through a second group. Time is going by. Now the patient is scheduled three months down the road. This is a guy who’s on his fourth visit with me. He’s got an area of necrosis and clearly not improving. I set up an MRI and my nurse calls the podiatrist’s office to see if he can get in any sooner― nope. I was thinking about getting vascular involved, and it was a bit of a run around, when the MRI demonstrated osteomyelitis. I ended up admitting him, and he got amputated. It was really a sad outcome. I feel like if there was better coordination, it could have been prevented.

This quote exemplifies the extent to which timely referrals are dependent on professional connections rather than systematic processes. While the study participant was new to a healthcare system, and therefore lacked professional connections within it, the quote also typifies difficulties faced by established rural providers when referring to urban specialists outside their healthcare system. The lack of professional connections bridging health systems is best evidenced by the fact that the vast majority of rural providers could not name a single urban specialist to whom they had referred a patient with a DFU.

### Tenuous connections between rural providers and urban specialists

All rural providers retained patients with DFUs in rural health systems when feasible: “We try to do as much as we can within our clinic setting prior to referring patients.” For example, rural PCPs often managed ulcers complicated by osteomyelitis, with one commenting “I guess that would be our responsibility.” This mirrored the sentiments of patients and caregivers, the majority of whom preferred to receive care locally. This preference was based not only on convenience, as it took considerably less time to drive to visits, it was also rooted in close personal connections that developed over time. One caregiver described sending a Christmas card to their podiatrist. Another explained “My daughter-in-law said ‘I don’t know how you live in a small town. Everybody knows your business.’ And I said, ‘I live in a small town *because* everybody knows my business.’” She was referring to a strong sense of community, where all members, including healthcare providers, could be trusted to help when needed.

While both rural PCPs and specialists preferred to manage patients locally as much as possible, they did refer to urban specialists outside the healthcare system when necessary. Typically, vascular surgeons were consulted to manage ischemic complications. Some rural providers referred to urban infectious disease physicians when cultures demonstrated challenging resistance patterns. Regardless of what triggered consultation, rural providers were faced with three main barriers: 1) time-consuming referrals, 2) negative experiences calling urban specialists, and 3) multiple, separate electronic health records (EHRs).

#### Time-consuming referrals

Nearly all PCPs expressed concern that the standard referral process would not result in timely consultation for patients with DFUs. Providers described that they generally ordered a consult, and a referral coordinator or scheduler placed the referral. This involved checking which specialist the patient’s insurance would cover and then communicating with that specialist’s office to relay the referral and supporting documentation. Administrative staff lamented the lack of a “work flow” for navigating referrals and authorizations with different health systems and insurances, stating such an algorithm would “save a lot of time.” This was a particular burden for rural clinics, which were located on the periphery of a number of health systems that often appointed different specialists to spearhead DFU care. One rural scheduler described the process as “a constant battle trying to find a place in a timely fashion.. . I place the phone call, beg and plead.” Once an appropriate specialist was identified, the specialist’s office would then call the patient to schedule an initial consultation. They inconsistently communicated back to the referring providers regarding the timing of that appointment. Most PCPs disliked this process. One stated, “I always worry. What happens if the ball gets dropped?” Another said, “Appointments can fall through the cracks.” A third described, “constant problems and mistakes, and people aren’t hearing back.” Patients and caregivers also expressed frustration with the length of time it took to be seen. One rural patient stated, “I called [an urban specialty clinic] in January. The earliest they could get me in was the first week of April. Really? You’re telling me that she’s booked for the next four months?”

Because of the urgency, providers placing formal referrals often expedited the process by communicating directly with specialists. This came at a cost. The time rural PCPs spent facilitating consultations detracted from their ability to provide intensive medical management for patients with DFUs. No PCP described diagnosing an ulcer and then being able to address glycemic control, smoking cessation, or optimizing medical management of vascular disease during that visit. Facilitating specialty care also overshadowed these tasks to the point where few PCPs scheduled follow-up visits to address them. However, they acknowledged that intensive medical management fell within their purview and was important for wound healing.

#### Negative experiences calling urban specialists

Rural providers sometimes telephoned urban specialists to facilitate urgent, outpatient consults. However, they did not have previously existing, professional connections in which to ground these discussions. While the vast majority of conversations went well, only one negative interaction was needed to derail collaboration. Some negative experiences were subtle: “[Urban specialists] are busy, and sometimes you get the sense that, unless the patient is really sick or you’re considering admitting them, they don’t necessarily want to take your call.” Others were overt: “[If] I’m referring a case to a tertiary care centre and they rip me up one side and down the other, then I just say, ‘Thank you very much,’ and find somebody else.”

#### Multiple EHRs

All providers lamented difficulties with multiple, disjointed EHRs spanning different healthcare systems. These poor connections were described as “disastrous.” EHR barriers included: having to remember separate user names and passwords for different systems; not knowing when or where to look for information; and incomplete sharing of information between systems that were linked. An inability to find information across EHR systems led to duplicate testing, difficulty triaging incoming referrals, and difficulty acting on specialty recommendations. Duplicate testing included repeating haemoglobin A1Cs, but also repeating expensive procedures. A rural wound care specialist recalled a patient who had ankle-brachial index testing three days apart in two different health systems. Some vascular surgeons reported repeating this testing because only the numeric indices were available through the linked computer systems, but waveforms were needed for clinical decision-making.

Poorly accessible information made referrals from different health systems difficult to triage. One urban scheduler noted, “Internal [referrals] tend to be easier because all of the imaging and testing is within our system and we can just pull it up. We don’t have to request anything extra. If it comes from an outside facility, we have to have [supporting documents] faxed.” Referral forms seldom included lists of desired information, leaving non-clinical staff to decide what to fax when records were requested and EHRs were not linked. An infectious disease clinic manager said, “When you have clinical staff like nurses and doctors working with non-clinical staff like receptionists [to place the referral and send records], that’s where the issues sometimes happen. I don’t expect non-clinical staff to understand the same things that a doctor or a nurse would.” Rural clinics were often under-staffed or lacked a formal medical records department, so that when specialty clinics did call for additional information, the transfer of supporting documents was slow. One vascular surgeon remarked, “By the time you get the records, you’ve usually already seen the patient [and told him/her] ‘We’ll talk to your provider and give you a call,’ So I feel that [getting the outside records] is the hardest, and it’s probably a long way off, but one computer system would be amazing.”

Barriers created by multiple EHRs directly impacted the ability of other providers to act on specialists’ recommendations. Rural PCPs described that the emergence of EHRs shifted the culture of consultation. Prior to widespread EHR use, part of the specialist’s role was to ensure the PCP was informed of his or her recommendations. Now, one PCP commented, “Their job is to document what they do and it’s my job to get in there and find it.” Most specialists still tried to send referring providers their notes electronically, but they were unclear how this process worked or if their notes truly reached their destination in a timely manner. One urban podiatrist noted, “We don’t even know if [the PCP] saw our note, so we don’t know if they’re considering our recommendation.. . The recommendation that we made two weeks ago hasn’t happened yet because of the inefficiencies.” Multiple EHRs consistently inhibited professional collaborations, especially between rural and urban providers. A rural PCP stated “I don’t feel like it’s a hostile relationship [with an urban specialist], I just feel like there’s been a little bit of a challenge of communication. More so because they’re on a different computer system so I can’t see what they did.”

### Outreach models for traditionally urban specialists

We interviewed two urban specialists who participated in rural, outreach care. While we did not reach saturation on this topic, we think these participants’ experiences offer valuable, initial insights into this burgeoning field. One participant was an infectious disease physician who provided predominantly inpatient telemedicine consultations. The other was a vascular surgeon who staffed in-person clinics. Neither system was described as optimal, but both were beneficial. In terms of telemedicine, consultation was consistently available for rural, hospitalized patients. The telemedicine physician had an excellent working relationship with the local podiatrist. They had done simultaneous exams and exchanged cellular phone numbers to facilitate care of shared patients. However, outpatient collaboration was problematic, as evidenced by a lack of interaction with rural PCPs:

That is a piece of this that is completely absent. This is a hole in multidisciplinary care. I don’t know A) how the PCP knows that a patient of theirs has been admitted, and B) how they’re able to view, or if they’re able to view, the inpatient record of what happened. . . I have not personally ever spoken to a PCP for a patient with a diabetic foot ulcer. I don’t have access to whatever treatments or evaluations they have done in the outpatient setting. So things I don’t know: what type of off-loading mechanisms were employed, what kinds of vascular imaging was obtained, A1C assessment, those kinds of things. And they may be just as much in the dark as to what I have done.

In contrast, the vascular surgeon who participated in outreach clinics did endorse close professional ties to some rural PCPs. However, he also noted that this was highly dependent on logistics: “In [rural clinic 1], I am working right next door to several primary care providers and just walking by the door they will wave, we will chat, maybe talk about a patient that they have seen or sent. In [rural clinic 2], I am in the basement of the hospital with no one around. Because of that I rarely have contact with any of the providers.” The vascular surgeon did not have an inpatient presence; rural patients he saw in outreach clinics needed to receive revascularization procedures in urban, tertiary care settings. Therefore, he felt that this outreach model could not meet all the needs of his patients.

### The value of multidisciplinary care and teamwork

Providers consistently agreed that multidisciplinary care was ideal for patients with DFUs. One PCP recalled, “I’ve seen multiple bad ulcers and cases of amputations, so I just know from experience that an interdisciplinary approach is really key to having that healing process actually take place. And it’s not straightforward. It doesn’t go well a lot of the time. So it makes me apprehensive, and it makes my patients apprehensive.” Another remarked, “I would argue that quality has to do with teamwork.” Participants agreed that multidisciplinary teamwork was enhanced by “open, good communication,” and professional connections. Connections that fostered future collaboration could be relatively modest. One podiatrist asserted that interactions which were “certainly face-to-face, even over the phone or voice-to-voice, versus my medical assistant talking to their medical assistant” helped build trust and served this purpose, regardless of the health systems in which providers practiced.

## Discussion

Ours is the first study to identify health system factors that may be driving the rural disparity in major amputations due to DFUs. Specifically, tenuous connections between rural providers and urban specialists were described as the main health system barrier to care. These connections stymied the initial referral process and subsequent, multidisciplinary collaboration. Furthermore, they impacted some of the highest risk patients― those with ischemia and complex infections― supporting an explanatory hypothesis for rural outcome disparities.

Effective integration of specialty care into DFU treatment is critical to the success of the currently espoused care model. Expert opinion guidelines suggest a tiered approach based on ulcer severity [20]. In this model, PCPs are responsible for preventing foot ulcers. Relatively straightforward ulcers can be managed locally, with collaboration between rural PCPs and rural specialists. Patients with advanced ulcers, especially those complicated by ischemia or deep infection, optimally are referred to a multidisciplinary limb salvage team at an urban, tertiary care system. If a dedicated team does not exist, referrals to individual urban specialists ensue. In either case, rural patients with advanced ulcers receive multidisciplinary care, but it spans two or more healthcare systems, which, in turn, challenges effective teamwork. Such challenges are less likely to be faced by multidisciplinary teams caring for urban patients, many of whom receive primary and specialty care within the same health system.

**Fig. 2:**
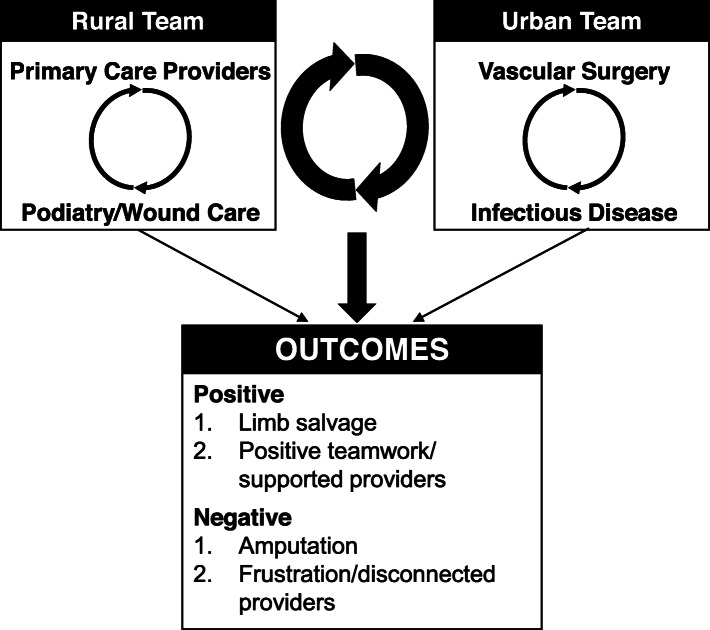
The rural-urban multiteam system caring for rural patients with diabetic foot ulcers. Human factors engineering emphasizes that coordination across teams (larger arrows) predicts success better than coordination within teams (smaller arrows). Our data identify poor connections across rural and urban teams as a contributor to rural disparities for patients with diabetic foot ulcers

Human factors engineering conceptualizes the care model that is used to treat rural patients with DFUs and spans at least two healthcare systems as a multiteam system [20, 21]. Specifically, rural PCPs and podiatrists or wound care specialists function as one team, managing glycemic control and local wound care. Urban vascular surgeons and infectious disease specialists function as another team, managing vascular disease and advanced infection. Together, they form a larger, multiteam system with the collective goal of ulcer healing (Fig. 2). This goal is dependent on the successful management of the four physiologic factors (glycemic control, wound management, vascular disease, and infection) within and across the urban and rural healthcare teams. Human factors engineering has confirmed that multidisciplinary teams spanning different systems require an added, overarching layer of coordination for optimal performance [21, 22]. Applied to DFUs, additional coordination is necessary, but currently lacking, for rural patients receiving care that bridges rural and urban systems. Rural providers were able to draw on professional connections to facilitate local collaborations. However, these professional connections were almost entirely absent when one provider was located in a rural system and the other practiced in an urban setting. Both locally and across the rural-urban divide, healthcare systems were not well designed to facilitate multidisciplinary collaboration needed for the optimal care of patients with diabetic foot ulcers.

Lastly, engineering research on multiteam systems has demonstrated that coordination across teams (i.e. coordination between rural and urban health systems) predicts success better than coordination within teams (i.e. coordination within either the rural or urban health system; Fig. 2) [23, 24]. Therefore, we hypothesize that focusing on improving connections between rural and urban healthcare teams (systems) should have a strong, positive impact on DFU outcomes. These efforts may also translate to other disease states with rural disparities, because the barriers and their potential solutions are unlikely to be limited to DFUs. For example, Noyes and colleagues identified similar barriers in coordinating cancer care delivery across rural and urban health systems in the United States [13].

Despite the strength of sampling many stakeholders, our study has limitations. First, we interviewed providers who were working in for-profit, American Midwest healthcare systems. Those working in universal healthcare systems, or those in different geographic regions, may face different challenges. The challenges bridging rural and urban healthcare systems are likely to be exacerbated by geographic isolation or workforce shortages. Contrary, patients receiving treatment in healthcare systems with strong network ties, or those with less dispersed referral options, may fare better. Second, our study solely focused on health system factors contributing to the rural disparity in major amputations. We chose this aspect because, as health system researchers, we are best poised to address it. However, our participants consistently mentioned socioeconomic factors, such as poverty and low education, as important to understanding the rural health disparity in major amputations. Third, we did not reach saturation among specialists that were based in urban healthcare systems but also provided rural, outreach care. Numerous institutions use telemedicine or teleconferencing to bridge the gap between rural and urban healthcare systems [12]. As an extension of this body of work, we suggest subsequent studies explore outreach models, including how they could be leveraged, and potential pitfalls, when caring for patients with DFUs.

## Conclusions

In conclusion, improving referral pathways and collaborations bridging rural and urban health systems is a promising way to address the rural health disparity faced by patients with DFUs. Furthermore, tenuous connections between rural and urban providers is unlikely to be disease-specific. Improving these connections may also help mitigate other rural health disparities where urban, specialist involvement is common.

## Supplementary information

**Additional file 1.**

## Data Availability

The datasets generated or analyzed during this study are not publically available due to our IRB restrictions in place to protect study participant identities, but they are available from the corresponding author on reasonable request.

## Abbreviations

DFU: Diabetic foot ulcer
EHR: Electronic health record
PCP: Primary care provider
RWHC: Rural Wisconsin Health Cooperative
WREN: Wisconsin Research and Education Network
