# Supplementary material for: Expect delays: poor connections between rural and urban health systems challenge multidisciplinary care for rural Americans with diabetic foot ulcers
Source: J Foot Ankle Res. 2020 Jun 16;13:32. doi: 10.1186/s13047-020-00395-y (PMC7278184; doi:10.1186/s13047-020-00395-y)
Supplement: Supplementary file 1 — Additional file 1. [file 13047_2020_395_MOESM1_ESM.docx]

**PCP Interview Guide**

Multidisciplinary teams, where people work side-by-side at tertiary care hospitals, have been shown to reduce the risk of major amputation for patients with diabetic foot ulcers. This is a good urban model, but it probably wouldn’t work well in rural settings. We are interested in learning about how we can modify the rural healthcare system, and perhaps how rural and urban healthcare systems interact, to promote care that addresses all the needs of rural patients with diabetic foot ulcers.

1. With this in mind, tell me about how you care for your patients who have diabetic foot ulcers.

“I diagnose” or “I manage” or “I prescribe”

Provider:

1. What aspects of care are you most comfortable handling? (ask this way, probe more if you need to)

Glycemic control

Infection

Offloading/wound care

Vascular disease

Home health or wound care nurse coordination

1. What aspects of care are more of a stretch for you?

What do you in cases like that?

Under what circumstances do you refer patients to different services (physicians and non-physicians)?

Why do you choose those particular ones?

Appropriate Referrals

1. What kind of access do you have to other types of providers that might be helpful (podiatrist, orthotics, vascular, ID)?

Are they in your health system/available/accessible?

How far away if they aren’t close?

How do you deal with crossing health system lines?

1. Walk me through how you refer a patient. What information is exchanged in this referral?

What information is included or needed?

Does this happen in person? On the phone?

Is this a formal or informal process (“I call my friend” vs. “I put in a formal referral for consultation…”)

What information do you or other specialists expect to have when you make or receive a referral?

1. What other ways, besides formal referral, do you use to involve other disciplines?

For example, do you seek an opinion even if another provider won’t see the patient?

How are you communicating? Through the EHR, on the phone?

Appropriate Response to Referrals

1. When you work with other providers to take care of these patients, what helps build trust between you all?

Examples might be: their presence/accessibility, concept of territorialism or “stepping on another provider’s toes”, understanding which provider is responsible for which aspect of care, fear or comfort in asking questions to other providers.

1. What makes for a good response from the provider that you referred the patient to?

What do you think is an appropriate amount of time to wait for a patient to see a consulting physician?

What do you think is an appropriate amount of time to hear from the consulting physician?

Is it clear how they are expected to respond to you or keep you informed? (examples include ability to see patient right away or soon, when the provider answers the question, understanding where to find results from tests or notes the other provider made)

1. What causes conflict with other providers?

(Examples from ID: misconception that if a patient sees ID, that they will receive antibiotics that day, patient priority or severity, wound healing)

1. How have you addressed conflict?

To other providers and/or to patients. Patient misconceptions can also be addressed.

1. What often gets overlooked?

Patient prompts:

1. What makes it easy to care for a patient with a diabetic foot ulcer?

Patients and providers. (E.g. when other providers are available, when a patient is accommodating/compliant)

1. What makes it difficult to care for a patient with a diabetic foot ulcer?

E.g. comorbidities, multiple health systems, long travel distances? Compliance?

Visit:

1. What happens in a typical visit?

Who rooms the patient?

What labs or tests are ordered?

Who else might be present?

How to follow up/refer?

1. How could the visit flow be changed to help?

With the EHR (note templates), putting in orders, etc.

System: If you had the power to change anything associated with this care…(be creative, innovative, thinking broadly..eg in a perfect world…)

1. What kinds of things do you wish happened automatically for you when managing a patient with a diabetic foot ulcer?
2. What kinds of things do you wish you could delegate, or pass off to someone else on the team?
3. What would this (automation/delegation) look like?

Wrap up: We would like to interview non-PCPs who you work with to care for these patients. Would you mind giving us a few names of people who might be interested in participating in our study?

Lastly, we will also be recruiting patients toward the end of the study. Can we hang recruitment flyers at this clinic?

**Non-PCP Clinician Interview Guide**

Multidisciplinary teams, where people work side-by-side at tertiary care hospitals, have been shown to reduce the risk of major amputation for patients with diabetic foot ulcers. This is a good urban model, but it probably wouldn’t work well in rural settings. We are interested in learning about how we can modify the rural healthcare system, and perhaps how rural and urban healthcare systems interact, to promote care that addresses all the needs of rural patients with diabetic foot ulcers.

1. With this in mind, tell me about the work you do for rural patients with diabetic foot ulcers.

“I diagnose” or “I manage” or “I prescribe”

Provider:

1. What aspects of care are you most comfortable handling?

Glycemic control, infection, offloading/wound care, and vascular disease

1. What aspects of care are more of a stretch for you?

What do you in cases like that?

Under what circumstances do you refer patients to different services (physicians and non-physicians)?

Why do you choose those particular ones?

Appropriate Referrals

1. Walk me through how you refer a patient. What information is exchanged in this referral?

What information is included or needed?

Does this happen in person? On the phone?

Is this a formal or informal process (“I call my friend” vs. “I put in a formal referral for consultation…”)

What information do you or other specialists expect to have when you make or receive a referral?

1. How is the PCP involved in this referral process?

Maybe consider inpatient vs. outpatient scenarios (PCP vs. hospitalist, or PCPs that still do hospital work)

1. What other ways, besides formal referral, do you use to involve other disciplines?

For example, do you seek an opinion even if another provider won’t see the patient?

How are you communicating? Through the EHR, on the phone?

Appropriate Response to Referrals

1. When you work with other providers to take care of these patients, what helps build trust between you all?

Examples might be: their presence/accessibility, concept of territorialism or “stepping on another provider’s toes”, understanding which provider is responsible for which aspect of care, fear or comfort in asking questions to other providers.

1. What makes for a good response from the provider that you referred the patient to?

What do you think is an appropriate amount of time to wait for a patient to see a consulting physician?

What do you think is an appropriate amount of time for you to hear from the consulting physician?

Is it clear how they are expected to respond to you or keep you informed? (examples include ability to see patient right away or soon, when the provider answers the question, understanding where to find results from tests or notes the other provider made)

1. Are there some common, wrong assumption about your practice that other providers make?

(Examples from ID: misconception that if a patient sees ID, that they will receive antibiotics that day, patient priority or severity, wound healing)

1. How do you deal with those wrong assumptions?

To other providers and/or to patients. Patient misconceptions can also be addressed.

1. What often gets overlooked?

Patient prompts:

1. When is it easy to care for a patient with a diabetic foot ulcer?

Patients and providers. (E.g. when other providers are available, when a patient is accommodating/compliant, comorbidities, communication, etc.)

1. When is it difficult to care for a patient with a diabetic foot ulcer?

E.g. comorbidities, multiple health systems, long travel distances? Compliance?

Visit:

1. What happens in a typical visit?

Who rooms the patient?

What labs or tests are ordered?

Who else might be present?

How to follow up/refer?

1. How could the visit flow be changed to help?

With the EHR (note templates), putting in orders, etc.

System: If you had the power to change anything associated with this care…(be creative, innovative, thinking broadly..eg in a perfect world…)

1. What kinds of things do you wish happened automatically for you when managing a patient with a diabetic foot ulcer?

Examples: paperwork/dictation

17. What kinds of things do you wish you could delegate, or pass off to someone else on the team?

18. What would this (automation/delegation) look like?

Wrap up: We would like to interview non-PCPs who you work with to care for these patients. Would you mind giving us a few names of people who might be interested in participating in our study?

Lastly, we will also be recruiting patients toward the end of the study. Can we hang recruitment flyers at this clinic?

**Patient interview guide**

We are working with healthcare teams, their patients and caregivers to come up with a better way to care for patients that get a diabetic foot ulcer and who need to be seen by their primary care provider as well as specialists (like podiatry, wound care, vascular surgery, infectious disease). We are interested in learning about your experiences with diabetic foot ulcers and talking about ways to make care better by getting your feedback and suggestions on some ideas our team has come up with.

**Part 1. Experiences with DFU’s**

You told me on the phone that you have/had a diabetic foot ulcer X years ago, **can you walk me through your experience** starting with when you first noticed something was wrong?

Probes:

1. How did you get to appointments? (Car, Abbivan, caregiver)
2. How did that go?
3. Often more than one provider needs to help. Did you have any experience with that? Who?

How did that go?

Can you give examples of multiple providers working together? (or not?)

How do you know who to call for what?

1. I’ve heard that when people are diagnosed with an ulcer, they all of a sudden have a lot of information thrown at them, about things like the wound, diet, smoking, et cetera. Is this something you can relate to?

How did you prioritize what to focus on?

1. Can you tell me about the healing process? Who else was involved?

Did you see or have you seen a diabetes educator?

Was that helpful? Why or why not?

Did a home health nurse come to your home? How did that go?

Was that helpful? Why or why not?

Smoking (Who should address this? (PCP, nurse, specialist, all, none; what resources would you like to have? I.e. counseling, accessibility for medication/gum)

What was difficult/easy? (frequency of appointments)

1. Walk me through the process of getting the appointment with X specialist set up.

Phone calls, mychart messages, etc.

1. What was it like getting day-to-day tasks done while your ulcer was healing? Did you need help? Who helped you?

Did you ever have to keep off your foot for an extended period of time? Can you talk about that experience? What were the biggest challenges overall?

What were the biggest challenges caring for the ulcer and your health?

1. What resources were available in (your community/town) to help you? (ADRC, etc.)
2. In the context of having an ulcer that requires multiple appointments with several providers, what has been your experience with health insurance?

Did you always have all the information you needed?

Who can you ask if you have questions?

1. Was there anything about getting your ulcer treated that surprised you?
2. Based on your experience, what could be improved?

What do you wish you’d had more help with?

1. What advice would you give a friend or family member if they were to develop a diabetic foot ulcer, having been through that yourself?

**Part 2. Strategies:**

Now I’d like to talk about some **ideas that we’ve come up with to help make care better**.

12. One idea that providers have is to make sure visits are scheduled before you leave the clinic. That way you would know when and where your next appointment is, regardless of whether it is with your primary care provider or a specialist like podiatry. What are your thoughts about that?

How would that go?

13. Let’s say you needed to travel to an urban clinic or center, such as Madison, to see a specialist and the specialist wanted imaging or testing done prior. Would you prefer to have the images or tests done in your local community before the appointment or at the center where your appointment will be on the day of the appointment with the specialist or before? Why?

ABI (ankle-brachial index) where there’s a blood pressure cuff on your arm and another on your ankle and the technician uses an ultrasound machine to look at your blood vessels.

Labs

Imaging (x-rays, MRIs)

14. Some clinics have what is called a case manager or patient navigator at their clinic. Typically, this is a nurse who helps coordinate appointments when people see multiple doctors like the podiatrist, endocrinologist, and primary care doctor. They also sometimes help with questions about insurance. Would you want to have access to a person like that? Why or why not? Does anyone help you like that now?

15. What would you think about a provider taking a picture of the ulcer, or you taking a picture of the ulcer, to help facilitate care?

Would you be willing or able to take the picture and send it to a clinic?

What concerns do you have about photos as part of care?

What types of concerns do you have about privacy or confidentiality with the photos?

16. Do you have any additional comments to make for the interview?

(Turn off microphones and give participant their compensation)

Now that you’ve participated in this study, our team is wondering if you would be interested in being contacted for future research opportunities with us?

If **no**, then say “Okay. Thank you for your participation in this study, and have a great day.”

If **yes**, then say “Great! Just so you know, your agreement to be contacted does not bind you into participating in future studies, nor does it ensure your participation. All this means is that when another opportunity arises, we will contact you. Your decision will not affect your medical care. After today, you can opt out of being recontacted at any time by contacting our study team or informing us in the event that we recontact you that you are no longer interested in participating in future research. Sound good? With that, how would you like to be contacted in the future, phone or email? Can you please confirm the phone number/email address? We will not share any contact information you provide, and it will be stored in a secure location only available to the study team.”

**Caregiver interview guide**

We are working with healthcare teams, their patients and caregivers to come up with a better way to care for patients that get a diabetic foot ulcer and who need to be seen by their primary care provider as well as specialists (like podiatry, wound care, vascular surgery, infectious disease). We are interested in learning about your experiences with diabetic foot ulcers and talking about ways to make care better by getting your feedback and suggestions on some ideas our team has come up with.

**Part 1. Experiences with DFU’s**

You/your spouse told me on the phone that the person you care for has had a diabetic foot ulcer X years ago, **can you walk me through your experience** starting with when you/your spouse first noticed something was wrong?

1. What was your role in the process? How did that go?

Driving

Helping with dressing changes (can the other person see the bottom of their feet?)

Attending vs. chauffeuring appointments

IV antibiotics at home

Diet for diabetes

Coordinating/keeping track of appointments (attending appointments)

Arranging the home to help with offloading

1. Often more than one provider needs to help. Did you/your spouse have any experience with that? Who?

How did that go?

Can you give examples of multiple providers working together? (or not?)

How did you know who to call for what?

1. I’ve heard that when people are diagnosed with an ulcer, they all of a sudden have a lot of information thrown at them, about things like the wound, diet, smoking, et cetera. Is this something you can relate to?

How did you prioritize what to focus on?

1. Can you tell me about the healing process? Were you the only caregiver, or did you need help? Who helped?

Did your person see or have you seen a diabetes educator?

Was that helpful? Why or why not?

Did a home health nurse come to your home? How did that go?

Was that helpful? Why or why not?

Smoking (Who should address this? (PCP, nurse, specialist, all, none; what resources would you like to have? I.e. counseling, accessibility for medication/gum)

What was difficult/easy? (frequency of appointments)

1. Walk me through the process of getting the appointment with X specialist set up.

Phone calls, mychart messages, etc.

1. What was it like getting day-to-day tasks done while your person’s ulcer was healing? Did you need help? Who helped you?

Did you ever have to help your person stay off their foot for an extended period of time? Can you talk about that experience? What were the biggest challenges overall?

What were the biggest challenges caring for the ulcer and your partner’s (husband/wife) health?

1. What resources were available in (your community/town) to help you? (ADRC, etc.)
2. In the context of having an ulcer that requires multiple appointments with several providers, what has been your experience with health insurance?

Did you always have all the information you needed?

Who can you ask if you have questions?

1. Was there anything about getting your ulcer treated that surprised you?
2. Based on your experience, what could be improved?

What do you wish you’d had more help with?

1. What advice would you give a friend or family member if they were to develop a diabetic foot ulcer, having been through this experience?

**Part 2. Strategies:**

Now I’d like to talk about some **ideas that we’ve come up with to help make care better**.

12. One idea that providers have is to make sure visits are scheduled before a patient leaves the clinic. That way you would know when and where your next appointment is, regardless of whether it is with your primary care provider or a specialist like podiatry. What are your thoughts about that?

How would that go/what would that look like?

13. Let’s say your person needed to travel to an urban clinic or center, such as Madison, to see a specialist and the specialist wanted imaging or testing done prior. Would you prefer to a) have the images or tests done in your local community before the appointment or b) at the center where your appointment will be on the day of the specialty appointment or before? Why?

ABI (ankle-brachial index) where there’s a blood pressure cuff on your arm and another on your ankle and the technician uses an ultrasound machine to look at your blood vessels.

Labs

14. Some clinics have what is called a case manager or patient navigator at their clinic. Typically, this is a nurse who helps coordinate appointments when people see multiple doctors like the podiatrist, endocrinologist, and primary care doctor. They also sometimes help with questions about insurance. Would you want to have access to a person like that? Why or why not? Does anyone help you like that now?

15. What would you think about a provider taking a picture of the ulcer, or you taking a picture of the ulcer, to help facilitate care?

Would you be willing or able to take the picture and send it to a clinic?

What concerns do you have about photos as part of care?

What types of concerns do you have about privacy or confidentiality with the photos?

If done before: How do they send pictures in? How does the physician know the pictures are there? How quick does provider respond?

16. Do you have any additional comments to make for the interview?

(Turn off microphones and give participant their compensation)

Now that you’ve participated in this study, our team is wondering if you would be interested in being contacted for future research opportunities with us?

If **no**, then say “Okay. Thank you for your participation in this study, and have a great day.”

If **yes**, then say “Great! Just so you know, your agreement to be contacted does not bind you into participating in future studies, nor does it ensure your participation. All this means is that when another opportunity arises, we will contact you. Your decision will not affect your medical care. After today, you can opt out of being recontacted at any time by contacting our study team or informing us in the event that we recontact you that you are no longer interested in participating in future research. Sound good? With that, how would you like to be contacted in the future, phone or email? Can you please confirm the phone number/email address? We will not share any contact information you provide, and it will be stored in a secure location only available to the study team.”
